# *Lived Lives: A Pavee Perspective*. An arts-science community intervention around suicide in an indigenous ethnic minority

**DOI:** 10.12688/wellcomeopenres.11330.1

**Published:** 2017-04-13

**Authors:** Kevin M. Malone, Seamus G. McGuinness, Eimear Cleary, Janis Jefferies, Christabel Owens, Cecily C. Kelleher

**Affiliations:** 1 Department of Psychiatry, Psychotherapy and Mental Health Research, UCD School of Medicine, St. Vincent’s University Hospital, Dublin 4, D04 T6F4, Ireland; 2 Centre for Creative Arts and Media, Galway Mayo Institute of Technology, Galway, H91 T8NW, Ireland; 3 Department of Visual Arts and Computing, Goldsmith College, University of London, London, SE14 6NW, UK; 4 University of Exeter Medical School, Exeter, EX1 2LU, UK; 5 School of Public Health, Physiotherapy and Sports Science, University College Dublin, Dublin, D04 E378, Ireland

**Keywords:** Suicide, Suicide Intervention, Irish Travellers, The Travelling Community, Suicide in Ethnic Minorities, Arts-science, Socially Engaged Art Practice.

## Abstract

**Background:** Suicide is a significant public health concern, which impacts on health outcomes. Few suicide research studies have been interdisciplinary. We combined a psychobiographical autopsy with a visual arts autopsy, in which families donated stories, images and objects associated with the lived life of a loved one lost to suicide. From this interdisciplinary research platform, a mediated exhibition was created ( *Lived Lives*) with artist, scientist and families, co-curated by communities, facilitating dialogue, response and public action around suicide prevention.

Indigenous ethnic minorities (IEMs) bear a significant increased risk for suicide. Irish Travellers are an IEM with social and cultural parallels with IEMs internationally, experiencing racism, discrimination, and poor health outcomes including elevated suicide rates (SMR 6.6).

**Methods: **An adjusted *Lived Lives *exhibition, *Lived Lives: A Pavee Perspective* manifested in Pavee Point, the national Traveller and Roma Centre. The project was evaluated by the Travelling Community as to how it related to suicide in their community, how it has shaped their understanding of suicide and its impacts, and its relevance to other socio-cultural contexts, nationally and internationally. The project also obtained feedback from all relevant stakeholders. Evaluation was carried out by an international visual arts research advisor and an independent observer from the field of suicide research.

**Results: **Outputs included an arts-science mediated exhibition with reference to elevated Irish Traveller suicide rates. Digital online learning materials about suicide and its aftermath among Irish Travellers were also produced. The project reached its target audience, with a high level of engagement from members of the Travelling Community.

**Discussion:** The *Lived Lives* methodology navigated the societal barriers of stigma and silence to foster communication and engagement, working with cultural values, consistent with an adapted intervention. Feedback from this project can inform awareness, health promotion, education and interventions around suicide and its aftermath in IEMs.

## Introduction

Suicide is the second leading cause of death in 15–29 year olds globally, and suicide prevention has been described as a “global imperative” by the World Health Organisation ^1^. However, despite it being a significant public health concern, there is still a knowledge vacuum in relation to this loss of life. In addition to epidemiological methods, suicide research has relied heavily on the psychological autopsy method to advance knowledge of at at-risk groups, and this method has been described as the cornerstone of suicide research ^2^. It involves gathering detailed information about a suicide death using a number of sources, including interviews with bereaved next-of-kin or other key informants, medical records and coroner reports. This comprehensive process allows for a “reconstruction of the lifestyle and personality of the deceased”, as well as adding to the understanding about the circumstances and events that may have led to their death ^3^. Many psychological autopsy studies have used a case-control design that allows for various risk factors for suicide to be better identified ^4^. Psychological autopsies can also provide insights into the experiences of families who have been bereaved by suicide ^5^, similar to “lived experience” research, with some studies citing therapeutic benefits of participation ^6^.

Research to date has focused more intensely on individual factors associated with suicide risk, and on clinical rather than community-orientated approaches to prevention. A recent study has identified that since so many suicide deaths occur at the “index” or first attempt (up to 60% in young males), greater efforts should be applied to earlier detection of at risk groups and to community interventions ^7^. Research has also relied heavily on psychiatric and social science methods to the exclusion of arts and humanities. The value and effectiveness of arts-science collaborations has become increasingly evident, with many well-established research institutions, such as Europe’s particle-physics laboratory CERN and the Wellcome Trust, establishing programmes to encourage such research. These collaborations facilitate researchers to look at their area of interest in a new way and think of novel approaches, providing “the ideal opportunity for interdisciplinary conversations” and public and community engagement ^8^. Arts and science collaborations have the opportunity to embrace all the components of the lived experience in their research inquiry.

**Figure 1.**
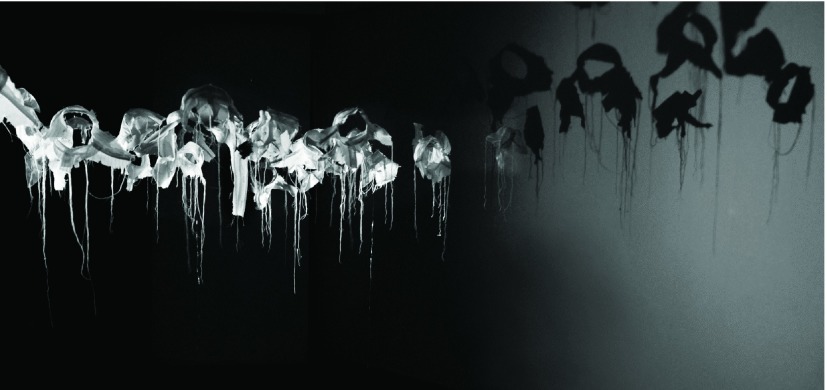
*21g (2003)*. McGuinness, S. (2015), Photo credit: Robert Ellis.

This study has its genesis in a coming together of an active clinical suicide research programme and a visual arts suicide researcher, who were both interrogating national suicide statistics within their respective disciplines. McGuinness had created a visual representation of young male suicide in Ireland in 2003 entitled *21g (2003)*, which consisted of an excess of 92 fragments of cloth (shirt collars) suspended from threads, one for each young male death that year, and each one weighing exactly 21 grams, the mythical weight of the human soul ( Figure 1). Meanwhile, the clinical scientist (KMM) was actively researching clinical and psychobiological risk factors for suicidal behavior, including suicide ^9, 10^. Professional paths converged in 2004 around the common goal of pursuing new knowledge and understanding about suicide, which led to a durational arts-science collaboration and the original research study *Lived Lives.*

*Lived Lives*, which in turn informed the present study *Lived Lives: A Pavee Perspective*, combined a psycho-biographical autopsy study (a modified psychological autopsy involving both quantitative and qualitative methods) together with novel “Visual Arts Autopsy” (VAA) methods ^11^, which are described in brief below. The study was approved by the St. Vincent’s Healthcare Group Ethics and Medical Research Committee, and with the full permission of the participating research families, the results of the study were brought into the public domain as a written document (Suicide in Ireland Survey 2003–2008) ^12^, a PhD thesis ^13^, and a series of arts-science mediated art experiences situated within the *Lived Lives* artworks ^14– 16^. Working in collaboration with many others, these arts-science mediated exhibitions have manifested in a series of public venues in Ireland, and the work has been presented at several international conferences in the UK, China, Canada and the USA ^a– e^. Feedback from these national and international manifestations of the project suggested that the *Lived Lives* project may transcend cultural boundaries and have the ability to engage marginalized and hard to reach communities, where suicide levels are known to be elevated.

Irish Travellers (also known as The Travelling Community) are one such group with elevated suicide rates. Irish Travellers are a distinct indigenous minority with many social and cultural parallels with other such groups internationally (e.g. Inuit, Indigenous Australian and Maori peoples) including elevated suicide rates and other poor health outcomes ^17^. The All Ireland Traveller Health Study (AITHS) ^18^ identified that over 11% of all Traveller deaths are related to suicide, with a 6.6 standardized mortality ratio (SMR). Elevated levels of frequent mental distress (FMD) were also recorded in 11.9% of respondents, and prevalence increased with age. Following adjustments for age and sex, FMD was more prevalent in those whose quality of life was impaired by physical health, by those who were recently bereaved of a friend or family member (frequently suicide-related), and by those who had greater experiences of discrimination ^19^.

Irish Travellers have a rich and creative cultural tradition, but also experience racism and discrimination ^20^, and were found by The European Parliament Committee of Enquiry on Racism and Xenophobia to be one of the most discriminated-against ethnic groups in Ireland ^21^. Irish Travellers have previously been recognized in British (including Northern Ireland) law as an ethnic group. However, their legal status in Ireland was that of a “social group” until March 2017, when they were finally officially recognized by the Irish State as an indigenous ethnic minority. An ethnic group is defined as one whose members identify with each other, usually on the basis of a presumed common genealogy or ancestry. Ethnic identity is also marked by the recognition from others of a group's distinctiveness and by common cultural, linguistic, religious, behavioral or biological traits. Traditionally, their nomadic lifestyle and limited education and literacy made them "hard-to-reach". Nowadays they are frequently under-served and excluded from health research, confounding intervention ^22^.

The aim of this project was to overcome the societal barriers of stigma and silence about suicide in Irish Travellers and to generate deep engagement and trust through dialogue, inclusion and co-partnership with arts, science and the Travelling Community, through the integrated arts-science *Lived Lives* practice. The project was supported by a Wellcome Trust Science and Society People award. Working with Pavee Point, an organization that has worked on a local, national and international level to develop innovative and culturally appropriate responses to the needs of members of the Traveller and Roma communities in Ireland using community development and partnership-based models, gave the project team the valuable opportunity to engage members of the minority population. Following intensive collaboration with members of the Travelling Community young and old, and international advisors over a year, the project *Lived Lives: A Pavee Perspective* manifested over a one week period (November, 2015) in Pavee Point, the National Centre for Traveller and Roma ^23^, and is the focus of this paper.

## The *Lived Lives* project

The methods for *Lived Lives: A Pavee Perspective* were informed by the original *Lived Lives* study, as described elsewhere ^11^. Briefly, the original *Lived Lives* project included a combined and integrated science-arts methodology, which involved the scientist (KMM) and the artist (SMcG) travelling to 104 suicide-bereaved families in their homes throughout Ireland to learn about lived lives lost to suicide (2003–2008) ^12^. The psycho-biographical autopsy study used a blended semi-structured interview and open narrative approach to document clinical, social and psychobiographical data on individuals lost to suicide, as related by their loved ones/next of kin. The VAA simultaneously invited families to donate images and other material belongings pertaining to the deceased, from which McGuinness created several *Lived Lives* artworks including *The Lost Portrait Gallery*, *Archive Rooms* and an iteration of *21g,* with 104 shirt fragments, 86 male, 18 female, one for every deceased included in the autopsy study (n =104).

*The Lost Portrait Gallery* consists of 39 jacquard tapestry portraits of the young suicide deceased. These jacquards are woven, worked from donations of snapshots and memorial cards donated by the families and friends to the *Lived Lives* archive. They are a photographic representation in cloth of the deceased. Each jacquard measures 36 × 22cm and is installed at exactly the height of the deceased individual.

*Archive Rooms* consists of clothing, writing and other personal objects belonging to the deceased, donated by the participating families, which were originally exhibited as a series of rooms with each loved lost one being represented by their individual belongings. Following the creation of these artworks, a series of site-specific experiential installations of these works and public conversations, mediated by the artist and scientist, were co-created and presented with the participating families' consent and active engagement, all of which was documented on video and with bereavement support present. This “ *Lived Lives*” public engagement model has been utilized in both urban ^a^ and rural ^e^ community manifestations.

## Lived Lives: A Pavee Perspective

### Building relationships and trust: Affective labour

The methodology for working with the Travelling Community in this study ( *Lived Lives: A Pavee Perspective*) drew on the design from the public installation of the *Lived Lives* artworks in Letterkenny (2013). This participatory and experiential community event was mediated by artist-scientist and had many elements, including a series of pre-planning consultations within the community, and with local health promotion and suicide prevention policy makers. As in previous public manifestations, the mediated exhibition consisted of a narrated walk for participants through the works, with the artist (SMcG) and the scientist (KMM) describing the *Lived Lives* project and the associated artworks and impacts. In Letterkenny, *Lived Lives* was open four days a week for four weeks for members of the public, which included visits from over 100 transition year school students from whom feedback was obtained ^e^.

*Lived Lives: A Pavee Perspective* was also informed by the novel methodology that has received international peer-review acclaim used in the aforementioned AITHS. The AITHS was the first study of its kind to examine the associations between socio-demographic and lifestyle factors with poor mental health in Irish Travellers. *Lived Lives: A Pavee Perspective* adapted AITHS methods that involved Irish Traveller peer researchers working on the project, taking leadership and co-ownership.

Collaborative pre-planning with leaders and groups within the Travelling Community began in September 2014. A pre-planning consultation and collaboration forum was established and representatives from Pavee Point leadership (social, health and youth) met with the *Lived Lives* team. The *Lived Lives* model was presented and discussed in detail and the rationale and pathway towards materializing *Lived Lives* with the Traveller tradition was outlined and agreed.

A follow-up pre-planning collaborative meeting took place on a number of Irish Traveller sites as part of an effective strategy to engage hard-to-reach communities. This involved the project team visiting Irish Traveller “halting sites” in the greater Dublin area where some members of the Community live in caravans, often in very unsafe environments from a health and safety perspective, with limited access to water, heating and electricity, and are significantly marginalized from society. Members of the Travelling Community met with the team at these sites and spoke of their complaints and concerns about health and safety often being ignored by various government organizations and official bodies. Trust was established by affording the Travellers an opportunity to be heard, on their own terms and in their own environment. The project team also travelled to Pavee Point for all follow up planning meetings. The Community Health Workers who facilitated the site visits and follow up planning meetings played a key role in this trust-building. Their mutual trust, respect and strong links with families within the Travelling Community allowed the researchers greater access and opportunities for engagement and collaboration for the project.

Workshops took place in Pavee Point in July 2015 with young Irish Travellers and their youth workers to include their voice in the process. This involved two groups of youths discussing the issue of suicide in their community and what they felt might be contributing factors and possible solutions. The young Irish Travellers were invited to respond to two questions in particular concerning suicide in their communities: “What can we do to stop suicide in our community?” and “What can the Traveller organizations do to stop suicide in our community?” Young Irish Travellers openly expressed their opinions and thoughts on suicide. When discussing what they felt they could do to stop suicide in their community, the most popular answers were: “talk to your friends, parents, brothers and sisters about how you're feeling”, “go to events that raise awareness about Traveller suicide”, “keep yourself occupied; get involved in healthy activities like sports”, “get involved with local youth services” and “involve parents and friends in awareness raising”. When discussing what Traveller organizations could do to stop suicide in the Travelling Community the most frequent answers were: “organize events to talk about suicide and mental health, especially with young people”, “go out to sites to talk to people about suicide”, “have people on sites trained in things like ASIST [Applied Suicide Intervention Skills Training] and Safe Talk”, “encourage and help Travellers to access mental health organisations” and “train Traveller youths in job skills, like the community employment scheme in Pavee Point”. These answers that emerged were used to create the new artwork of *The Hawthorn Tree*, described below.

All pre-planning collaboration was documented on video and in still image. Some concerns about documenting the community event (about image and confidentiality) were raised during the pre-planning phase, as many Travellers expressed negative prior experiences with the media. Fears as to how the footage would be used were addressed through an agreement that there would be a show-back prior to public dissemination.

### Tailoring and adapting Lived Lives as key to community engagement

**Figure 2.**
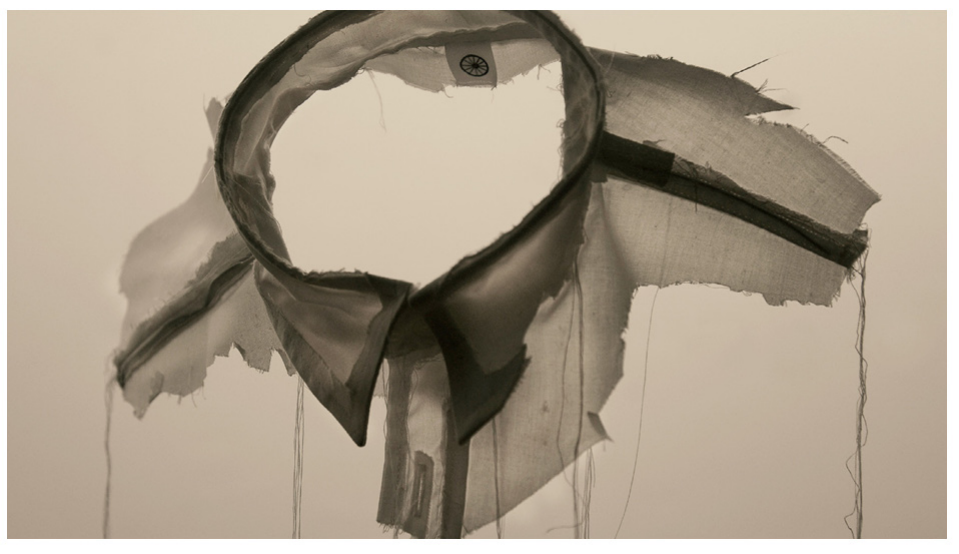
*21g 6.6*. McGuinness, S. (2015), Photo credit: Robert Ellis.

Members and representatives of the Travelling Community openly engaged with the *Lived Lives* project team, and it was agreed to aim to host *Lived Lives* in Pavee Point in autumn 2015. In preparation for the event, there was a further twelve meetings between project partners to decide what information would be displayed and which issues were most relevant to be discussed over the week-long installation. Simultaneously, McGuinness worked on adjusting the physical form of the *Lived Lives* artworks and recasting “ *21g”* to have relevance to the Travelling Community. The artworks were recast to both the physical site of Pavee Point, a non-traditional exhibition venue, and also to reflect the issue of elevated Traveller suicide rates. The recast *21g 6.6* (the *Pavee Perspective* version) contains 76 shirt collars, each weighing 21g, the mythical decline in body weight at the precise moment of death. A total of 66 of the shirt fragments had a small printed wagon wheel stitched to the collar ( Figure 2), representing male Traveller suicide rates. The remaining 10 collars had no identification marks and represented the rest of the male population in Ireland *. 21g 6.6* is a visual representation of SMR for Irish Traveller suicide death rates, questioning the elevated male Traveller suicide rates in particular.

**Figure 3.**
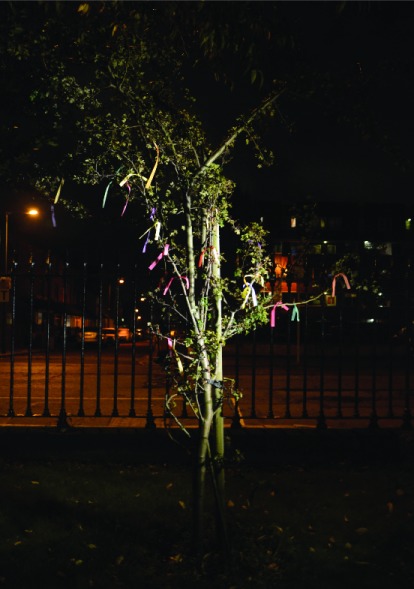
*The Hawthorn Tree*. McGuinness, S. (2015), Photo credit: Robert Ellis.

The most popular responses from the youth group discussions were printed on cloth ribbons and Irish Traveller audiences of the *Lived Lives* event were subsequently invited to choose the response that they most identified with and tie their selection to a hawthorn tree planted in front of Pavee Point, as part of a new artwork *“The Hawthorn Tree*” ( Figure 3). The Hawthorn tree has significance amongst the Travelling community dating back to an old custom of “rag trees” where Travellers believed that if a piece of clothing from someone who is ill, or has a problem of any kind, is hung from the tree (usually a Hawthorn tree and usually near a Holy Well) the problem or illness will disappear as the rag rots away. Sometimes the rag would represent a wish or aspiration that will come to pass as the rag decays.

### Lived Lives as a community event

A week prior to the exhibition, the *Lived Lives* team moved into Pavee Point building and installed the recast works. The *Lived Lives* exhibition and mediated experience was hosted over a seven day period at Pavee Point. Similarly to the manifestation in Letterkenny, each day of the project involved mediated group tours of the works, which included the *21g 6.6, Lost Portrait Gallery, Archive Rooms,* and a number of short research films that documented the engagement of the bereaved *Lived Lives* families with each other and the artworks **.** These were followed by facilitated round table discussions with each group, often amid the physical artworks, where there was an opportunity to discuss reactions to the exhibition and share thoughts about the issue of suicide within the Travelling Community. The exhibition was open to the public and we also invited suicide prevention policy makers, clinical directors from mental health services, funders, medical students, art students, suicide bereaved families and members of the Travelling Community of all ages to participate throughout the week. In total, 150 members of the Travelling Community and 34 members of the general public engaged throughout the week (120 female, 64 male). Bereavement support was available throughout the event, as per the ethics protocol. The entire event was documented on both still and video camera with a proposed show back to participants after the event and before public dissemination, with a right of participants to withdraw their image/contributions if they so wished. These filmed discussions would later be used as feedback on the project and were of particular importance with respect to Irish Travellers to overcome any literacy issues (a point noted in relation to the oral visual technique used in the AITHS to collect information).

The opening night of the project took place on Friday, November 6 ^th^ and included attendance from the Travelling Community, *Lived Lives* families, *Lived Lives* project team, All Ireland Traveller Health Study investigators and invited guests. Introductory welcome and remarks were contributed by Michael Collins (an Irish Traveller Youth Worker), Ronnie Fay (Director of Pavee Point), Kevin Malone, Seamus McGuinness and special guest actor John Connors, who is a member of the Travelling Community and also a strong advocate for Traveller health promotion following his own lived experience with mental health and suicide bereavement (see Opening Night Video). A member of the Travelling Community, Helen Hutchinson also performed a reading of a poem she wrote about suicide death in Travellers entitled “The Traveller Pain”, which she enunciated amidst the *21g 6.6* installation ^24^.

The following day was a closed event for suicide-bereaved Irish Traveller families and for any members of the original *Lived Lives* families who chose to attend. The families all shared a mediated journey through the works followed by an engaged roundtable discussion around the issue of elevated suicide rates within the Travelling Community. This format continued throughout the week with enduring conversations between Irish Travellers and the previously mentioned other groups (bereaved families, including *Lived Lives* donor families, medical students, art students, policy makers from the domains of clinical intervention and suicide prevention, senior clinical decision makers, arts and cultural policy, funders). The unique interaction between members of the Travelling Community and members of official bodies opened up new conversations and discussions about suicide intervention and prevention (see Walking Through the Lived Lives Works Video, Irish Traveller Conversations about Lived Lives Video and Reflections of Irish Travellers and Policy Makers Video).

### Participant feedback on the community event

In addition to the facilitated discussions, everyone who engaged with the exhibition was also invited to share their feedback via a short written semi-structured questionnaire if they wished. This questionnaire contained six questions, which were modified from previous manifestations of *Lived Lives* and had yes/no response options and space for further qualitative comments that were later thematically analyzed.

A total of 81 attendees (63 females, 18 males), with a mean age of 38 years, voluntarily filled out the *Lived Lives* feedback questionnaire, of which 33 identified as Irish Travellers. A summary of the qualitative results can be seen in Table 1. As the questionnaire was also open-ended, some participants chose to give qualitative feedback also. “Moving”, “poignant” and “powerful” were common words used to describe the experience of *Lived Lives: A Pavee Perspecti*ve. One participant (male, 30) described it as “Very powerful. Personalizes suicide, makes it a story, not a statistic”, while another (female, 19) said “I found the exhibition powerful. It’s heavy but it needs to be to get the point across”. Participants also described the exhibition as encouraging conversations about suicide that perhaps might otherwise not have taken place: “I think this exhibition opens up a space where conversations can be had about the effects of suicide without it been [sic] overshadowed by any feelings of stigma” (female, 46), “helps make you more open about talking about and discussing suicide which is so important” (female, 43). Another theme that emerged from the data was that *Lived Lives* portrays the devastating effects of suicide on bereaved families and friends: “families lives ruined” (female, 34), “sense of pain of families and friends left behind” (female, age not stated), “it’s the heartbreak left behind” (female, 60), “very poignant work. Brings a real tangibility to the lives passed, the invite to touch and feel and engage removes the taboo about suicide. It breaks the distance somewhat between the worlds of ‘I or my family have never been affected by this’” (female, 45).

**Table 1.** Feedback summary for *Lived Lives: A Pavee Experience*.

Question ( *n=79)*	Yes	No	Don’t know	No answer
1. In your opinion, is Lived Lives an effective means for addressing the subject in the Traveller community in Ireland?	71 (90%)	1 (1%)	5 (6%)	2 (3%)
2. In your opinion, do you think Lived Lives should be open to and seen by all ages?	68 (86%)	6 (8%)	5 (6%)	0
3. Do you think that Lived Lives 'glamourizes' and/or 'romanticises' suicide'?	1 (1%)	72 (91%)	3 (4%)	3 (4%)
4. Do you think that Lived Lives could be an effective means of suicide prevention or ways of helping people following a suicide death within the Traveller Community?	68 (86%)	1 (1%)	10 (13%)	0

### Integrated feedback from observers

Evaluation was an integral part of the project, and an expert longitudinal evaluator from the field of visual arts (JJ), who was intimately familiar with the project since its inception in 2006, attended for three days. An independent expert evaluator from the field of suicide research (CO) was present for the entire event. JJ was embedded in the mediated tours and round table discussions, while also taking part in the feedback sessions with the various groups of attendees, including the Irish Travellers, medical students and policy makers. The independent evaluator (CO) used an ethnographic approach, observing each of the events, moving among the different audiences, interacting with them and with the exhibition itself, questioning, reflecting and seeking to understand how the installation might achieve its aims of creating new knowledge and understanding about the elevated risk of suicide within the community. The independent evaluator also considered how the project may address the stigma and taboos surrounding suicide and overcoming cultural divisions between the Travelling Community and mainstream Irish society. Observations were collected in the form of extensive field notes, supplemented by the camera footage, and analyzed using a thematic approach informed by qualitative research methods. Oral reflective feedback was shared by evaluators at the end of the event followed by subsequent written evaluation. Following the event, both evaluators provided feedback reports on the project and its impacts from their professional perspectives ( Supplementary File 1 and Supplementary File 2).

### Reflection and outputs

The final day and phase of the project was a reflection day between all partners to discuss whether the project met its objectives, what went well and what perhaps could have been improved. An online digital publication was put together over the following months, which documented the project in detail through still and moving images, all of which were shown back to the participants for their views and input prior to online publication going live (May, 2016) and has been made available to Irish Travellers for education, health promotion and suicide awareness ^24^ through a dedicated *Lived Lives: Pavee Perspective* website http://www.apaveeperspective.com.

## Discussion

To our knowledge this is the first report of a research project that applied an arts-science collaboration to add to knowledge and understanding about suicide and its stigma in marginalized and hard to reach communities with elevated suicide rates. The novel methods employed yielded deep community engagement and reached its target audience.

### Engagement

*Lived Lives: A Pavee Perspective* demonstrates that hard-to-reach audiences can be reached and engaged on sensitive health issues such as suicide. Our experience has demonstrated that this has to unfold slowly and participants must be centered within the art-science collaborative process, having equal say in how their private experience can be transposed to wider publics. The project team were aware throughout the process of how past research in the Travelling Community had often lacked important ethical considerations and focused on problematizing Travellers, thus perpetuating negative stereotypes. The *Lived Lives* methodology encouraged inclusivity from the start and the Irish Travellers involved took co-ownership of the project and moved towards taking active steps to address the problem of suicide in their community. Irish Travellers who had seen the exhibition in the first couple of days often returned and brought others and gave a “tour” of the artworks themselves with their own personalized narration, taking co-ownership of the project, adding a real “Pavee Perspective” to the experience for everyone involved. The willingness of all participating audiences to be filmed created enduring outputs and gave a voice to members of the Travelling Community to discuss one of the biggest taboos in their community (as seen in videos linked above). Irish Travellers also articulated a desire to remain involved in *Lived Lives*, “be represented” in future exhibitions, and expressed a wish that other members of the Travelling Community around Ireland would have the opportunity to engage with the project in the future.

### Tailoring health promotion

The Irish Travellers who took part in the project articulated the need for tailored health promotion, intervention and suicide prevention. They expressed this need for male Irish Travellers in particular who encounter difficulties with traditional healthcare pathways and difficulties with help-seeking, suggesting that stigma around help-seeking may be sustaining elevated suicide rates in the Travelling Community.

Sweeney *et al*. reported how young men contemplating suicide may often send inconsistent signals to those around them, thus making it difficult for friends and family to judge whether intervention is needed ^25^. Sweeney *et al*. also acknowledge how individuals may be “too ready” to accept assurances from a friend that they will not take their life. Lay judgements of level of risk can often be influenced by whether the distress is viewed as an expected response to certain life events, such as a relationship break up. Lay judgement may be even more complex in the Travelling Community based on the heightened stigma of mental illness, suicide and help-seeking experienced by its members ^25^.

Many national suicide prevention strategies reference the needs of at risk communities, many specifically identify the suicide rates in indigenous ethnic minorities ^21, 26^, but none have specific guidelines concerning tailoring meaningful and effective interventions within communities, which are so-called “hard-to-reach”.

For example, Ireland’s national strategy to reduce suicide “Connecting for Life” identifies Travellers as a “priority group” with strategic goal 3 of the plan being “To target approaches to reduce suicidal behavior and improve mental health among priority groups” ^26^. Under this goal, the main objectives are: “Improve the implementation of effective approaches to reducing suicidal behavior among priority groups”, “Support, in relation to suicide prevention”, “the Substance Misuse Strategy to address the high rate of alcohol and drug misuse” and “Enhance the supports for young people with mental health problems or vulnerable to suicide”. The action steps to achieving these objectives center around “targeted” initiatives and services in primary care settings and schools, as well as collaborating with relevant organizations to design “targeted” policies and mental health programmes, particularly for youths. *Lived Lives: A Pavee perspective* is a real world example of a tailored and targeted initiative specifically for Travellers, which engaged the community across the life cycle around mental wellbeing and suicide.

### Lived Lives as an “intervention”?

**Figure 4.**
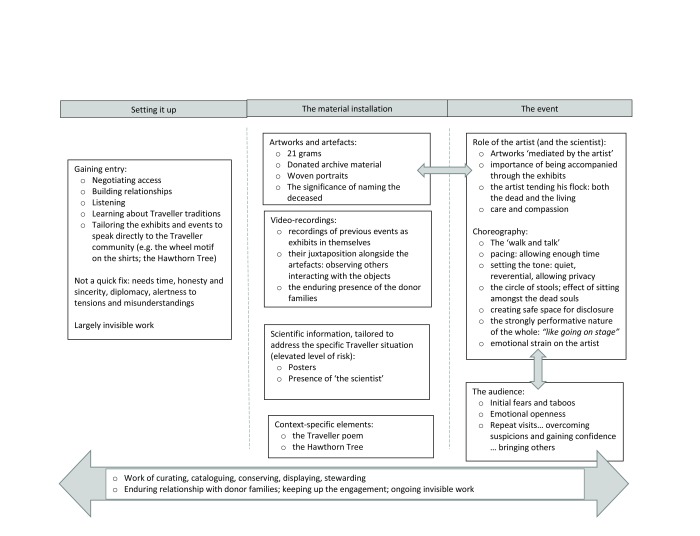
Core components of the “intervention”. Engaging the Irish Traveller Community with the reality of suicide through exhibition of a visual arts autopsy.

Questions remain as to whether or not *Lived Lives* can be considered “an intervention”. In Figure 4, the independent observer (CO) explores the core components that were involved in this particular “intervention”, based on close observation of the *Lived Lives: A Pavee Perspective* project over the course of the full week and listening to the accompanying discussions. These components include (i) the “largely invisible work” involved in setting up the project and engaging communities to take part; (ii) the actual material installation of the tailored-to-context artworks and information; and finally (iii) the event itself, in which the artist and scientist accompany different communities through the work and facilitate open dialogue and discussion around suicide within a caring and compassionate environment.

The *Lived Lives: A Pavee Perspective* project contains characteristics of all four types of community health interventions, as described by McLeroy *et al*. ^27^, by (a) being based in a community setting (Pavee Point being a community institution); (b) the Travelling Community themselves being the “target” of the intervention; and (c) the community also acting as a resource and (d) as an agent. The Travelling Community acted as a resource through their co-ownership and active participation, which McLeroy *et al*. describe as “essential for success in population-level health outcomes”. In terms of acting as an agent, the Irish Travellers examined, through their participation, the “natural adaptive, supportive and developmental capacities” of their community by bringing others along to the exhibition, delivering their own mediated tour of the artworks from their perspective, and discussing the need for change. The methods used also map the five key principles identified by Netto *et al*. for adapting behavioral interventions for ethnic minorities: (i) using community resources to publicize the intervention and increase accessibility; (ii) identify and address barriers to access and participation; (iii) develop communication strategies that are sensitive to language use and information requirements; (iv) work with cultural or religious values that either promote or hinder behavioral change; and (v) accommodate varying degree of cultural identification ^28^.

Suicide intervention can refer to any effort to bring about change to reduce suicidal behavior in individuals or within communities. In that sense, this project may have attributes of “an intervention”, as it enabled a stigmatized community, who rarely speak of suicide or mental illness, to open up, discuss and acknowledge the problem among their people. Despite these levels of stigma and resistance to speaking about suicide in the Travelling Community, the Irish Travellers observed that their engagement with *Lived Lives* “got them talking”, opening up and looking at the issue of suicide from a new perspective. Moreover, the project empowered Irish Travellers, policy makers, funders, clinicians, educationalists and suicide bereaved families to come together as equals with evidence of meaningful adjustment of attitudes and position, consistent with an intervention process.

Specific, objectively measurable outcomes are more difficult to identify. Throughout the week, subtle and quiet engagement and actions were happening at the margins of the installations. These included hushed conversations, whispers, religious gestures, embraces and tears. The discreet audio visual methodology allowed for these private and intimate moments to be documented and will become an integral part of future *Lived Lives* installations, in line with the expressed wishes of the Irish Traveller voice. Perhaps these observations suggest that intervention itself is an essentially subjective experience and typically requires re-assessment over time.

### Cultural context in suicide research

The results of *Lived Lives: A Pavee Perspective* highlight the importance of bearing cultural context in mind when doing suicide research. Cultural mistrust, cultural beliefs and traditions are key factors to be considered when designing suicide prevention and intervention initiatives for ethnic minorities. Historically, mental health professionals have neglected factors such as social support and spirituality as culturally relevant factors in service users’ ability to cope with psychosocial problems ^29^. This is of particular importance in relation to Irish Travellers, a community with particular religious and spiritual ties. The inclusion of medical students in the engagement of the project aimed to develop a deeper awareness of suicide and related cultural issues for the “doctors of tomorrow”. This engagement will be reported in detail elsewhere.

Although traditionally religion is considered to be a protective factor for suicide, the elevated Irish Traveller suicide rates would suggest otherwise. There are particular mourning rituals associated with death in the Travelling Community, such as the traditional wake, the ninth day after burial and the month’s mind mass, which is sometimes celebrated every month for the first year after a death ^30^. Traditionally, Irish Traveller custom was to burn the deceased’s home and belongings because the memories were too painful. Although not as common now, possessions are still often given away or destroyed, and if the deceased lived in a caravan it may be sold or the family may wish to move accommodation. This however is not always possible due to the many housing issues faced by the Travelling Community. These customs in relation to possession are a counterpoint to the *Lived Lives* project, where memories and possessions of the deceased are central. Suicide deaths are particularly challenging to cultures with strong religious beliefs, and participants in the project expressed how suicide deaths in the Travelling Community are often not spoken about afterwards, yet another barrier to be faced in overcoming stigma.

Irish Travellers bear similarity to Indigenous Australians in the frequency of deaths in their community. Poor health outcomes, infant mortality and suicide rates (often young adult males) mean that much of the life of Irish Travellers and other indigenous peoples are spent grieving lost loved ones. Tatz refers to this as a “perpetual cycle of grief”, describing it as a “constant cycle, or procession, of grief. There is no time to complete the grieving before another death ensues, and there is almost no grief counselling available” ^31^. The idea of perpetual grief among Irish Travellers adds to the story of suicide within the community, possibly contributing to the many clusters of young Irish Traveller suicide deaths that were anecdotally referred to throughout the project.

Irish Traveller engagement with a project that challenges some of their oldest and deepest superstitions and stigma possibly illustrates the urgency within the community to overcome their fears and address the issue of suicide. Their engagement also possibly illustrates the potential of *Lived Lives* to create a compassionate space that transcends traditional cultural boundaries. Feedback over its many public manifestations suggests it is a universal, non-judgmental, and tangible portrayal of suffering and loss. It also incorporates being present to the pain of others, acceptance of death by suicide and offers consolation around individual and collective grief *. Lived Lives: A Pavee Perspective* enabled Irish Travellers to speak about the unspeakable and acknowledge suicide death in their community. The mediated experience motivated an expressed desire amongst members of The Travelling Community to move beyond the cycle of grief, stigma and silence, to participate in the *Lived Lives* project and to take collective community action.

### Evaluation, feedback and limitations

Evaluation has been integral at all manifestations of the project. The methodology remains novel and thus there are few comparable studies from which to draw examples on how best to “measure” results from this kind of research, bearing subjectivity in mind. We chose to adopt an integrated and blended evaluation approach where we benefited from the collective wisdom of an embedded internal art research expert (JJ), and an external suicide and qualitative research expert (CO), as well as the expertise of the everyday voice.

Our project and subsequent write-up raises the challenge of disseminating non-traditional research projects. The study challenges the traditional structure for reporting research (the IMRD format), in which there is strict differentiation between methods and results. It is fluid, adapting and adjusting to communities, site and context. This raises the question of where, and in what format, arts-science collaboration results should be published and presented, and we have opted for a more blended science-arts reporting style in this paper. An evaluation of “Be Creative Be Well”, an innovative initiative in the UK that comprised around 100 different participatory art projects aimed at promoting health and wellbeing in London, framed the work “not as a piece of academic research but as an action-based inquiry that would produce an informative and thought-provoking report, the findings of which could usefully be shared with others” ^7^. It suggested ways in which public health professionals and artists can learn from each other in order to work more effectively both together and with local communities, “firmly” grounding their report in “the complexities, pressures and constraints of the workaday world”. We have provided all available images, videos and written documentation of the project, as well as interactions and discussions that took place throughout the event. This contrasts with a traditional report approach describing and analyzing the whole experience in any single way. We expect that through the open access portal a greater, more open evaluation phase may take place, which in turn will enhance the project’s dissemination and provide greater, richer feedback and analysis.

Traditionally, *Lived Lives* has always collected rich combined oral and written feedback. However, due to varying literacy levels within the Travelling Community, written feedback in this instance was limited, with only an estimated 40% of those who filled out the form identifying as Travellers. However, the opportunity to provide oral feedback (the Travellers have a strong oral tradition) addressed this issue for the most part, with many participants providing rich feedback orally on video during the round table discussion groups and through the mediated tour of the artworks.

This oral tradition is also in part why ethnographic methods were chosen as one of our primary evaluation methods. According to Joe *et al.*, priority should be given to qualitative ethnographic methods when researching suicide in groups with high associations of stigma ^29^. These methods may be more effective in capturing the complexity of the experiences of ethnic groups and what may be contributing to suicidal behavior among members. Joe *et al*. also suggest “ethnographic methods may be more culturally congruent with the preferred modes of communication”, which was evident in this project in relation to the oral tradition of the Travellers.

More female members of The Travelling Community than males participated in and attended the project. Women are traditionally more active in health promotion and awareness within their community, and in this case vowed to take the message back to their male counterparts. Similar to traditional Irish society, the Travelling Community is also traditionally patriarchal, and in order for effective change to take place an increased number of men may need to take active roles in promoting suicide prevention activities, especially given that the rates are most elevated amongst men. The lesser attendance of men may be related to the male help-seeking stigma in the Irish Traveller culture.

Although installing the *Lived Lives* project in Pavee Point Traveller and Roma Centre was very symbolic and demonstrated the commitment of the Travelling Community to the project, there may have been some practical drawbacks to the location. Netto *et al*.’s principles for adapting health promotion interventions include identifying and addressing barriers to access and participation. This refers not only to societal barriers, but practical barriers such as providing transport and keeping costs of participation low. Although youth groups were provided with both transport and times to attend the *Lived Lives* installation, the inner city location may have been inconvenient for many Irish Travellers living on the outskirts of the city. The timing of the event (10am–4pm) may also have had an impact on male attendance. As was demonstrated in the AITHS, successful health promotion initiatives within “hard-to-reach communities” are often supported by trust being established over long periods of time and the initiatives being led by members of the community themselves. Whilst we pursued this approach based on the AITHS methods, it may need to be more of a focus in future manifestations. Future manifestations will also consider “venue” as an important factor in ease of access, and greater efforts should be made to recruit male attendees and to facilitate their schedules, as we have evidence from other community *Lived Lives* engagements with several hundred young men that they actively responded to the project and gave specific positive feedback in this regard ^13^.

## Conclusions and future directions

This project created pathways for Irish Travellers to safely articulate the specific challenges they face as a community in relation to elevated suicide and mental health issues. It also facilitated dialogue about their specific needs as a marginalized community in Ireland (only recently officially recognized by the Irish State as an indigenous ethnic minority) in acknowledging, discussing and addressing these issues. Based on the levels of engagement in North Dublin with the project, we suggest a similar project may be effective in engaging other members of The Travelling Community around the country who face similar challenges and perhaps even greater challenges in the isolation of their locations. The online publication is designed to be accessed by Irish Traveler organizations throughout the country.

We also suggest the methodology is transferable to other indigenous ethnic groups globally. The stigma and challenges of addressing the issue of suicide among indigenous ethnic minorities, articulated by the Irish Travellers in Pavee Point, are mirrored globally in communities such as the Indigenous Australians, First Nations of Canada and Maori people of New Zealand. In addition to transferability to other ethnic minorities, the project illustrated its ability in overcoming obstacles associated with research with other hard-to-reach and stigmatized at risk groups for suicide, such as LGBT, prisoners and those with mental illness. “Reach out” is a common mantra in suicide prevention. Our project suggests perhaps by using novel arts-science methods and “reaching in” and empowering so called “hard-to-reach” communities, tailored interventions for at risk groups can be identified and evaluated.

## Ethics and consent statement

This research was approved by the St. Vincent’s Healthcare Group Ethics and Medical Research Committee (ethical approval number: R043) as part of the dissemination of the original *Lived Lives* Visual Arts Autopsy Study and Suicide in Ireland Survey (ethical approval number: R040).

All moving and still images, quotations and names included are done so with the consent of the event participants.

## Footnotes

^a^ *Suicide in Ireland: A Conversation and Journey through Loss with Science and Arts* Exhibition at Royal College of Physicians’ Ireland, Dublin, May 2010 ( http://www.inarchive.com/page/2011-09-09/http://www.rcpi.ie/News/Pages/RCPIPublicMeetingonSuicide.aspx

^b^ *Lived Lives; with Mary Jane Jacob, Seamus McGuinness, Joan Freeman and Prof Kevin Malone.* Exhibition Presented in Dublin Contemporary, Dublin, October, 2011. **

^c^Merriman Summer School on “ *Changing Irish Childhoods”* Opening Presentation, Dublin, August, 2011.

^d^ *Hangzhou Triennial Of Fibre Art Exhibition*, Presented at Hangzhou, Zhejiang,China, November, 2013.

^e^ *Lost Lived Lives Exhibition* Presented in Letterkenny Regional Cultural Centre, Letterkenny, Donegal, Ireland, November 2013.

## Supplementary material

Supplementary File 1: Evaluation by Janis Jeffries, Professor of Visual Arts and Computing, Goldsmith College, University of London (May 2016).

Supplementary File 2: Evaluation by Professor Christabel Owens, Associate Professor of Public Mental Health, University of Exeter Medical School, Exeter (June 2016).
