# Supplementary material for: Lived Lives: A Pavee Perspective. An arts-science community intervention around suicide in an indigenous ethnic minority
Source: Wellcome Open Res. 2017 Apr 13;2:27. [Version 1] doi: 10.12688/wellcomeopenres.11330.1 (PMC5439511; doi:10.12688/wellcomeopenres.11330.1)
Supplement: Supplementary file 1 [file wellcomeopenres-2-12230-s0000.tgz › d3a936a7-24e1-4eb6-98e9-7b1dd8093365.docx]

**Supplementary File 1:** **Evaluation by Janis Jeffries, Professor of Visual Arts and Computing, Goldsmith College, University of London (May 2016).**

In Together: The Rituals Pleasures and Politics of Cooperation (2012) Richard Sennett observes that we need to understand cooperation, collaboration and impact through the idea of the dialogical: “The subjunctive mood is most at home in the dialogical domain, that world of talk that makes an open social space, where discussion can take an unforeseen direction. The dialogic conversation... prospers through empathy, the sentiment of curiosity about who other people are in themselves.” (p.15)He argues dialogic conversation in terms of practical application; change requires the desire to find spaces and places: caring and trusting environments where there is time, respect for difference, and a generous capacity to listen to others whose ideas about creativity might be different from our own. The Lived Live project, which began in 2006 with a scholarship from the Ad Astra Foundation, UCD to fund an artist - Seamus McGuinness, from GMIT in Galway, Ireland – to pursue a practice research PhD in a long-term research project with scientist Kevin Malone, School of Medicine and Medical Science, University College Dublin. It was the first project of its kind internationally, and remains unique. The first phase of the research was completed in 2010, when McGuinness was awarded a PhD for this research, culminating in dissemination, exhibition and several public meetings in Irish society. I have been involved with the project since 2006 as one of the supervisors to the creative practice PhD and subsequently in various public engagements and evaluations. The exhibition, “Lived Lives: A Pavee Perspective, was the most public of all the public engagement activities so far and the most creative, engaging deeply and meaningfully with a hard to reach community, Irish Travellers. As we know, behind the statistics there are many untold stories of Lived Lives of those who have lost someone to suicide. The Lived Lives project is the consequence of conversations and research interviews with 104 suicide bereaved families, mainly focusing on young males who had taken their own life. New and experimental research methods, combining visual arts practice and scientific research, have enabled the production of new knowledge and understanding. It has been, in many ways, a co-collaboration with families bereaved by suicide, as is evident by their continued engagement with the project. The key question, then as now, is how can a creative arts practice (cloth, material objects, donated belongings and significantly stories of everyday experience) contribute to and extend our understanding of suicide and mental health? It is a big ask and a huge challenge. With support from the Wellcome Trust, McGuinness and Malone have spent the last year (2014-2015) working with Travellers, leaders and young people, thinking about how Lived Lives might have meaning for them. As a result, and as part of a series of ongoing conversations, Lived Lives: A Pavee Perspective was presented at the Pavee Point Travellers Centre in Dublin in November 2015.Irish Travellers are a traditionally nomadic group with origins in Ireland who possess a separate identity, heritage and culture to the settled community in general. An Irish Traveller presence can be traced back to twelfth century Ireland, with migrations to Great Britain in the early nineteenth century. The Irish Traveller community 118 Lived Lives: A Pavee Perspective (2015) is categorized as an ethnic minority group under the Race Relations Act, 1976 (amended 2000); the Human Rights Act 1998; and the Equality Act 2010. Irish Travellers are not recognized as an ethnic minority by the Irish State. Some Travellers of Irish heritage identify as Pavee or Mincier, which are words from the Irish Traveller language, Shelta. The Traveller communities are widely considered to be among the most socially excluded communities in the UK and Ireland. Life expectancy is much lower than that of the general population, with Traveller men and women living 10-12 years less than the wider population. Travellers have higher rates of infant mortality, maternal death and stillbirths than the norm. Travellers experience constant racism in the media and elsewhere, which would be socially unacceptable if directed at any other minority community. In addition, and for the purposes of “Lived Lives” at Pavee Point, the All Ireland Traveller Health Study (AITHS) (University of Dublin, 2010) documented a high rate of metal health illness and suicide within the Traveller community with suicide the case of 11% of all Traveller youth deaths, and 6.6% increased risk of suicide in Traveller men, most commonly between 15-25 years of age. Whilst Traveller men find it difficult to express their feelings, Traveller woman Helen Hutchinson wrote a moving poem, “The Traveller Pain” (2015), which is displayed opposite a further iteration of McGuinness’ 21g 6.6, a work remodeled, re-created and repurposed for Pavee Point. The Lived Lives installation at Pavee Point“21g” originally consisted of 104 white shirt collars and fragments hanging at eye level for viewers to walk through, around and under. The Pavee Point installation 21g 6.6 contains 76 shirt collars, each weighing 21g. 66 of the shirt collars with a stitched wheel symbolizing the Traveller community relate to Traveller men, the other 10 relating to settled men. Now raised to eye level, the installation reverberates with those walking though the treads and cloth, which touch the viewers’ body, leaving traces of the encounter on suit shoulders. What is important to note that is that an individual does not walk through in isolation, but is guided through the work and the stories of its production by McGuinness and/or Malone. Stools are gathered underneath “21g 6.6” and a dialogue emerges with those that have visited the whole exhibition, Travellers, members of the public, other artists and medical professional, officials and medical students as well as a special and closed session for the Lived Livesfamilies who travel from far and wide to view the Archive Rooms, the Archive Films and The Lost Portrait Gallery to share their experiences with others. Whilst social interaction is often thought of as technological, the significance of 21g 6.6 is that there is a different kind of immersion. It is not virtual, but intensely physical, as people whisper quietly or silently go into themselves to reflect on those too that they may have lost. The move from installation to the next staging of the work proposes a different set of actions and engagements. In Galway in 2009, at the private family viewing the Archive Rooms were separate, but now items donated by the families to the physical “Lived Lives” archives are mixed together (with their collective agreement). I noticed different objects and different relationships as a football shirt and entry ticket to a Manchester United game were positioned below a toy bicycle and a tin cooking pot. Photographs, drawings, poems, cries for help, guitars, and stethoscopes mingle with one another on wall shelves, opposite low level plinths with shirts and shoes. A three minute video of families entering their personal archive room from their first engagement in Galway ‘09 remind us of individual loss but shared pain in the new configuration of artifacts and memories of loss. This also unique in the sense that the pace of moving through one part of the exhibition to the next combines slow walking and intense moments of privacy peppered with conversation between visitor and visitor and their guide. By the time visitors enter the Lost Portraits Gallery, emotional viscerality has taken over any ‘objective’ evaluation. Removed from the white, marbled circular site of the Royal College of Physicians Dublin in 2010, 32 delicate Jacquard portraits, are rearranged in a one longish, cream-colored room, with a comfortable chair at the end for you to watch the jacquard weaving process on video. Just some of the portraits are shown, the sound of the loom overtaking the sound of any tears in the room. Each production scene begins with the name of the deceased before the face of the person comes into being. The Process of Engagement Firstly, cloth moves people: touch and tactility are embedded both in the work and in the viewers’ responses. Physical and material artifacts appear familiar and open up another space for dialogue. Secondly, the short video extracts from the roundtables, conversations and interviews with the Lived Lives families (2006-2010) collectively and individually show the slow evolutionary process of building trust over time between the artist and scientist as they build another, durational portrait of collaboration and co-operation. Thirdly, the physical objects and video works cannot be separated from the conversation that is the ‘final’ part of the viewing experience. For those who find communication difficult, sitting underneath 21g 6.6 enables a site for beginning to speak in a public, but also private setting for complex emotions to be phrased in words. Tales are told, stories unlocked, grief is shared, feelings released, voices are heard.
