# Supplementary material for: Lived Lives: A Pavee Perspective. An arts-science community intervention around suicide in an indigenous ethnic minority
Source: Wellcome Open Res. 2017 Apr 13;2:27. [Version 1] doi: 10.12688/wellcomeopenres.11330.1 (PMC5439511; doi:10.12688/wellcomeopenres.11330.1)
Supplement: Supplementary file 2 [file wellcomeopenres-2-12230-s0001.tgz › 38bbf3a6-b00c-429e-ae8a-399f516e2a69.docx]

**Supplementary File 2:** **Evaluation by Professor Christabel Owens, Associate Professor of Public Mental Health, University of Exeter Medical School, Exeter.**

**Aims of the evaluation**

- Experience the installation at first hand as an outsider
- Observe and document Travellers’ interaction with the exhibition (and with the project team)
- Describe core components of the ‘intervention’
- Identify possible outcomes and mechanisms of action, and contribute to the development of a programme theory.

**Methods**

The project incorporated a novel approach to evaluation involving the use of an independent expert observer. A social scientist and international expert in suicide prevention (Prof. Christabel Owens) was present at Pavee Point Traveller Centre throughout the week and used an ethnographically-informed approach, observing each of the events, moving among the different audiences, interacting with them and with the exhibition itself, questioning, reflecting and seeking to understand how the installation might achieve its aims of: a) creating new knowledge and understanding about the elevated risk of suicide within the

Traveller community; b) addressing the stigma and taboos surrounding suicide; c) overcoming cultural divisions between the Traveller community and mainstream Irish society. Observations were collected in the form of extensive field notes, supplemented by the camera footage, and analysed using a thematic approach informed by qualitative research methods.

This was methodologically challenging. Ethnography is the study of people in their natural setting. Traditional ethnographers immerse themselves in the everyday world of a particular group, in an attempt to see the world as they see it and to uncover the cultural rules and unarticulated beliefs and assumptions that shape their social reality.^1^ The Lived Lives project was different insofar as the setting was unnatural; what was being observed was no-one’s everyday social reality. The installation took the Travellers, the project team, the evaluator and other visitors out of their everyday comfort zones, challenging them all to see things anew. What was being observed was a collision between a number of very different worlds, a challenging of habitual and culturally normative ways of thinking and being, and a subtle process of change. Much of what was going on, as visitors moved among the exhibits, was of a private and internal nature and was therefore unobservable. There was a reverential hush during the walk-throughs. If people spoke at all, it was in whispers that were inaudible to the evaluator, who had to rely instead on picking up non-verbal signals (quiet tears; bowed heads; a Traveller woman crossing herself) and interpreting silences. In order not to break the mood, the evaluator made a conscious decision not to force people to articulate their feelings by asking questions, relying instead on simply being alongside them and sharing the experience, and on the power of intuition. Ethnography is always subjective, but the subjectivity was of necessity magnified in this case. It was also personally challenging. Whilst the camera team were always on the outside, silently looking in, the evaluator’s position was not clear. Was she a member of the audience, of the project team, of the camera team, or of all three at once? In practice, it required fluidity and an ability to move between insider and outsider roles depending on the context, and led to one or two awkward moments for everyone.

**Highlights and Key Observations**

The most enduring and powerful image is that of the artist, Seamus McGuinness, alone, moving quietly among the suspended torn white shirt collars of the 21g exhibit, carefully separating tangled threads and adjusting the angle at which each delicate shirt collar is hanging. So much that is central to the Lived Lives project is encapsulated in that image. It is almost biblical. It puts one in mind of The Good Shepherd tending his flock, knowing each one by name and keeping watch over it, which indeed is what McGuinness and Malone have done throughout this long-running project and continue to do during each new exhibition, extending the utmost care and compassion to both the dead and the living. Listening to them chatting with the donor families confirms their genuine and abiding interest in these families’ lives: *“And how’s [other child] doing?”*

The presence of both the artist and the scientist throughout the week was a vital ingredient. Promotional material and research protocols describe Lived Lives as an experiential art installation ‘mediated by the artist’. Each new visitor or group of visitors is escorted through the exhibition by McGuinness, who quietly explains the contents of each room, the rationale behind it and the creative processes involved, then allows them to look, read, touch, absorb and feel for themselves, privately, before judging when to move on. The ‘walk and talk’ is artfully choreographed: the pace of movement; the time allowed for quiet reflection; the amount of information given; the tone of voice. It is visual art, and also essentially performative. The unobtrusive but reassuring presence of the creators of the project, on hand the whole time, and the fact of being accompanied on one’s first encounter with the exhibits creates a sense of safety, which is essential. As one visitor said of the exhibition: *“You can’t just walk in off the street and think you’ll take a look at it. It’s big stuff, this.”*

What makes it “big stuff” is in fact the smallness and ordinariness of much of it. A pair of glasses, a scruffy notebook, a battered mug, a diminutive pair of Irish dancing shoes. These are not ‘works of art’. They are ordinary items, teenagers’ stuff, once taken for granted and in everyday use, now frozen in time. The ‘art’ lies in the recognition of their power to communicate. They connect one directly and viscerally with the deceased, with the homes they left behind and the gaping voids that opened up in those homes with their departure. It is impossible not to touch; to hold the items as the deceased would have done, to finger their woven portraits. Touching is central to the whole experience. It is why McGuinness works with textiles and cloth, materialising loss. Touching removes distance. Touching is also a two-way process: as we touch, so we are touched by the exhibits.

As we walk gingerly through 21g, allowing ourselves to be caressed by the torn shirt fibres, the gravity of it all remains in the abstract. Each shirt represents a lost life, but a nameless one. This exhibit is material, but still statistical in nature. In that respect alone, it is a safer place to start. Visually it is the most dramatic and arresting. Many of the Traveller community were noticeably fearful of walking amongst the absent bodies. *“It gives you the shivers,”* one young man commented to another. When we enter the Archive Room and Lost Portrait Gallery, statistics go out of the window, as does the ethical rule book. Here we get to know those who died, intimately, as individuals. Not only their names, but the size of their shoes, the perfume they wore, the team they supported, their favourite things. The minutiae of daily lives, once lived. In some cases, their very last words to their friends and families. At the heart of the Lived Lives project has been the desire to ‘restore identity to the deceased’; to reveal the real lives behind the statistics, the enormity of each loss. The naming of the deceased and the filming of the donor families – their identification - may be controversial, but it works. *“Rebecca will be on my mind all night,”* says a Traveller woman.

The silences, as each group moves through the exhibits, are deep and loaded, broken now and then as one Traveller reads aloud to another, in a low voice, the content of a suicide note. Contrasting sharply with this reverential hush during the walk-through is the babble that breaks out over the tea cups afterwards. Like the conversation that flows at the church door once the service is over, signifying a state of emotional release. There can be no question that the Lived Lives installation stirs powerful emotions. If that is what it is designed to do, it is certainly effective. Comments on the pink feedback forms are dominated by references to the power of the material exhibits to move: *“Sad, sad, sad”; “Very painful”; “Affecting”; “Heartbreaking”*. No-one leaves without having felt something of the pain of those who died, those they left behind and those who will be bereaved tomorrow and the next day and the next, until a way is found to stop the inexorable toll of suicide on society. Unlike a mainstream scientific exhibition, Lived Lives gives those who engage with it permission to feel, both their own and others’ pain. In doing so, and in holding them during the experience, it accomplished two things during the week at Pavee Point. It brought people together, Travellers and settled people, overcoming the sense of otherness, however temporarily, through recognition of the universality of grief and loss. ‘Their’ pain is the same as ‘our’ pain. Grief and loss know no cultural boundaries. They affect everyone alike. *“You don’t see a settled person; you see a person that’s lost their child,”* admitted one member of the Traveller community. Another Traveller woman stands in front of the red ball gown, crosses herself and sends up a silent prayer for the young girl who once wore it. It is reminiscent of the black and white images of Christmas Day 1914, English and German soldiers leaving their respective trenches to kick a football about together. A temporary lifting of the barriers of race, language and ideology; a putting aside of hate. On one pink form is written: *“A candle should be lit for them.”*

It also lifted the powerful taboo on talking about suicide, again perhaps temporarily, but a lifting nonetheless. Many comments on feedback forms refer to the urgent need to ‘open up’ and ‘speak out’ about suicide and its impact on families and communities. The donor families could be seen as having paved the way. There was recognition from Travellers of their courage in doing so, and a growing sense over the course of the week that Travellers could, and must, do the same: *“It’s a hard subject to talk about, but you’ve got to talk about it.”*

One of the most remarkable things to watch during the course of the week was a shift in the power dynamic that manifested itself suddenly and unexpectedly on the morning of Day 5. Up until then, each of the sessions had been orchestrated by McGuinness and Malone, the former presenting his artistic creations and the latter the scientific evidence. The Travellers had mainly held back, allowing control to rest with the ‘experts’. They came, they listened, they looked, engaging with the exhibits; they drank tea and talked amongst themselves. On the Tuesday morning, the fourth full day of the exhibition, they took control. The planned session with young Traveller groups, for whatever reason, did not happen. Instead, a large group of Traveller women assembled, some of them repeat visitors bringing friends and neighbours and escorting them through the exhibits, taking over from McGuinness and providing their own commentary. They then settled themselves round the table and, spontaneously, began to talk. Like women of old around a campfire, they told their own stories, narrated their own losses: *“I had a niece that did it. Mother of five children... She had everything going for her... Lovely young woman, she was.”* They talked of their worries about their menfolk and their young ones, lamenting a lost world in which *“... the children were never bored, they used to play the quoits and the marbles, the horseshoes and the like.”* Something shifted during that session, with the unlocking of the Travellers’ tales. They took ownership of the problem: *“It’s killing our people,”* and, by implication, it’s we who have to do something about it. *“We have to go back to our own groups now and talk about it.”*

One of the project’s initial questions was: Is the Lived Lives installation transcultural and universalizable? The Traveller women provide the answer. Of the week-long event, one says, *“It’s got us talking about it.”* It required extended exposure to the exhibition over the course of several days to erode the suspicion and fear with which they first entered and to build confidence. We must also not underestimate the amount of time, energy and commitment on the part of the project team that was required, not only to mount the exhibition in a physical sense, tailoring the exhibits to the Traveller context as they did so, but, prior to that, to gain access to the Traveller community at all. This up-front bridge-building activity was not witnessed by the evaluator, but it is clear that the event could never have happened without a huge investment of invisible – and affective – labour. What also became clear during the week was the emotional strain, particularly on the artist, that each ‘performance’ imposed. Lived Lives is far more than a collection of artworks and artefacts.

**Where next for the “Lived Lives” project?**

Despite the tailoring of exhibits to speak to the Traveller community that was done on this occasion, is there is a danger of its becoming fossilized or set in aspic? One is tempted to ask: Why these lives, and only these lives? The lack of cultural and ethnic diversity in the donor families was stark from the outset, but it was not until the Tuesday morning that the women felt able to say: *“I would like to see a Traveller family donate something to that room.”* Their strong desire to contribute and for Travellers’ ‘lived lives’ to be represented alongside those of the settled people is further evidence that a cultural divide has been bridged.

How might the project continue to evolve and to embrace other communities around the world, new lives, new media and forms of art, new artists, maybe even engaging bereaved families in the creative process, beyond the mere donating of items? These questions are still to be addressed.

**Where next for the Traveller community?**

Their questions remain:

How to get their men to talk about distress

How to know that someone is depressed or feeling suicidal; seeing the signs

How to access services when even non-marginalised groups fall through the gaps

How to bear the ongoing pain of living with discrimination, exclusion and rejection: “always hiding who you are.”

The ongoing challenge will be to help them to address these issues in a meaningful way.

McGuinness and Malone describe the Lived Lives visual autopsy project as being driven by a desire to delve behind the suicide statistics, bringing to the fore the identities of those who died. They have used artistic forms and the language of textiles to do this, allowing the deceased’s abandoned possessions and their woven images to speak. Owens and colleagues have long been engaged in the same endeavour, collecting narratives given by the bereaved and refusing to reduce their stories to statistics, by privileging qualitative methods over quantitative.^2-4^ Text versus textile; the verbal versus the visual. The journey is the same, as is the desired endpoint: to allow affect into the picture. In 2015, in a plenary paper delivered at the IASP World Congress, Owens called for a more emotionally-informed science of suicide prevention.^5^ Ever-rising suicide rates worldwide will never be reversed by reason alone. Lived Lives uses the language of the heart, and it is sorely needed.

**References**

1. O’Reilly J. Key Concepts in Ethnography. London: Sage; 2009.

2. Owens C, Lambert H, Lloyd K, Donovan J. Tales of biographical disintegration: how parents make sense of their sons' suicides. *Sociol Health Illn* 2008; **30**(2): 237-54.

3. Owens C, Lambert H. Mad, Bad or Heroic? Gender, Identity and Accountability in Lay Portrayals of Suicide in Late Twentieth-Century England. *Culture, Medicine & Psychiatry* 2012; **36**(2): 348-71.

4. Owens C, Owen G, Belam J, et al. Recognising and responding to a suicidal crisis in the family and social network: qualitative study *BMJ* 2011; **343**: d5801. doi: 10.1136/bmj.d5801.

5. Owens C. Preventing suicide at the micro-social level: Understanding the needs of concerned family members and friends. Plenary presentation at IASP World Congress. Montreal, Canada; 2015.
