# Supplementary material for: Lived Lives: A Pavee Perspective. An arts-science community intervention around suicide in an indigenous ethnic minority
Source: Wellcome Open Res. 2017 Apr 13;2:27. [Version 1] doi: 10.12688/wellcomeopenres.11330.1 (PMC5439511; doi:10.12688/wellcomeopenres.11330.1)
Supplement: Peer review history

# Peer review: Referee response for version 1

**DOI:** 10.21956/wellcomeopenres.12230.r21996

**Published:** 2017-05-16

**Authors:** Celia Lury

**Affiliations:** 1 Centre for Interdisciplinary Methodologies, University of Warwick, Coventry, UK

It is rare to see such a scrupulously documented and careful discussion of interdisciplinary collaboration. The approach and outcomes are described in detail, explicating how aims were achieved. The article also presents the nature of the collaboration in such a way that it can be adapted for further projects. It is an exemplary demonstration of the value of interdisciplinary collaboration.

I have read this submission. I believe that I have an appropriate level of expertise to confirm that it is of an acceptable scientific standard.

# Peer review: Referee response for version 1

**DOI:** 10.21956/wellcomeopenres.12230.r21909

**Published:** 2017-05-11

**Authors:** Susan Chatwood

**Affiliations:** 1 Institute for Circumpolar Health Research, Yellowknife, NT, Canada

Thank you for the opportunity to review this article, it was a pleasure to read. It addresses an important societal concern, and provides a clear and well justified example of interventions to address suicide in indigenous populations.

 The interdisciplinary approach is clearly described and provides detail sufficient to be replicated. The participatory methods are well designed and appropriate for this study. The partnerships developed for the project are well described and provide a strong example of interdisciplinary work between arts and science.

 The integrated and blended research approach can be difficult to document, the authors have done an excellent job in describing and justifying this approach, and specifically why it is beneficial to understand suicide in communities. 

 For Canada - indigenous peoples include Inuit, First Nations and Metis.

I have read this submission. I believe that I have an appropriate level of expertise to confirm that it is of an acceptable scientific standard.

# Peer review: Referee response for version 1

**DOI:** 10.21956/wellcomeopenres.12230.r21886

**Published:** 2017-05-03

**Authors:** Hyeouk Chris Hahm

**Affiliations:** 1 School of Social Work, Boston University, Boston, MA, USA

First, I applaud the incredible efforts that Dr. Malone *et al*. have made for Lived Lives project. I enjoyed reading this manuscript, and it is a beautifully written document which well illustrates the process and needs of this research.

 While studying community engaged suicide intervention, this project is innovative, creative, and powerful. As authors indicated in the manuscript, suicide intervention can refer to any efforts to bring about change to reduce suicidal behavior in individuals and within communities. Through having the arts-science collaboration and getting the bereaved families involved in this project, it opened up the door to discuss one of the most stigmatizing topics of human experiences: suicide. In order to reduce the suicidal behavior of the community, the community has to gain a collective understanding of the impacts and  the contextual factors of suicide as well as the targeted initiatives and policies that take the particular community’s specific and special needs into consideration.

 Furthermore, the majority of suicide prevention neglects “targeted” and “tailored” intervention and continues to assume “one size fits all policy.” Dr. Malone and Mr. McGuinness targeted “Irish Travellers”, the vulnerable, under-studied, and under-served population. They adapted their Lived Lives work to fit this community’s voices so that it can better foster the open conversation about the taboo topics around mental health, suicide, and barriers. They also aimed to raise the awareness of this community's problem (the perpetual cycle of grief) to the public as well as its own “Irish Travellers” community.

 After reading this manuscript, I can envision that Lived Lives project can be adapted to other ethnic minority populations (e.g, Asian-Americans, African-Americans) in any international countries or communities.

 However, there are several comments to improve this paper.

1. In the Introduction, I suggest that the information regarding “Irish Travellers” should come to the front. More statistics regarding the suicide rates and  mental health functioning data compared to their counterparts will strengthen the significance of this study.
2. On page 4, “Nowadays they are frequently under-served and excluded from health research, confounding intervention.”  I do not understand what the authors  mean by “confounding intervention”. It will be helpful if the authors can clarify this point by re-writing this sentence.
3. While the evaluation of the project is simple and crude, I recommend the authors to come up with a more rigorous evaluation process for the future Lived Lives work with other populations. I believe this part should be added in the future direction section.

I have read this submission. I believe that I have an appropriate level of expertise to confirm that it is of an acceptable scientific standard.
